# Design, Optimization and Fabrication of a 28.3 THz Nano-Rectenna for Infrared Detection and Rectification

**DOI:** 10.1038/srep04270

**Published:** 2014-03-06

**Authors:** M. N. Gadalla, M. Abdel-Rahman, Atif Shamim

**Affiliations:** 1 IMPACT Lab, Computer, Electrical and Mathematical Sciences and Engineering Division King Abdullah University of Science and Technology, Thuwal 23955-6900, Kingdom of Saudi Arabia; 2 Prince Sultan Advanced Technologies Research Institute (PSATRI), College of Engineering, King Saud University, Riyadh 11421, Kingdom of Saudi Arabia

## Abstract

The increasing energy demands of the world's population and the quickly diminishing fossil fuel reserves together suggest the urgent need to secure long-lasting alternative and renewable energy resources. Here, we present a THz antenna integrated with a rectifier (rectenna) for harvesting infrared energy. We demonstrate a resonant bowtie antenna that has been optimized to produce highly enhanced localized fields at the bow tip. To benefit from this enhancement, the rectifier is realized between the overlapped antenna's arms using a 0.7 nm copper oxide. The thin film diode offers low zero bias resistance of 500 Ω, thus improving the impedance matching with the antenna. In addition, the rectenna prototype demonstrates high zero bias responsivity (4 A/W), which is critical in producing DC current directly from THz signals without the application of an external electric source, particularly for energy harvesting applications.

The world has witnessed an unprecedented increase in its energy requirements over the last few decades driven by the increasing size of the population, industrial development and the increasing level of activity of humans around the globe. Consequently, new paradigms are needed to produce clean and cheap energy. Infrared (IR) energy harvesting from waste heat can be a promising contribution for sustainable energy. Waste heat vacillates between, approximately, 400 K and 2000 K (2–11 μm) and is produced by different sources such as metal heating and melting, steam generation, fluid heating, heat treating, and agglomeration^1^. Rectennas can be used for waste heat energy harvesting if the *RC* time constant of the rectifier is in the range of femtoseconds. In this case, rectenna is considerably colder than waste heat and can be used for DC electricity generation. Rectenna can also be used for waste heat reduction and cooling of electronic devices, taking advantage of their diminutive size that facilitates integration with electronic devices.

An infrared rectenna is a combination of a receiving nano antenna and a THz rectifying diode. Unlike photovoltaic where the conversion efficiency is limited by the semiconductor band gap, rectennas utilize the wave nature of light and can, theoretically, achieve 100% conversion efficiency. This statement, however, assumes perfect antenna metals, maximum reception by the antenna, and complete matching between the antenna and the rectifier. For a certain frequency of operation nano antennas must be optimized to produce the maximum field enhancement. The relatively high frequency of operation for THz rectennas leads to challenges not only in the fabrication of the nano antennas but also in the rectification process, since typical semiconductors based rectifiers do not have high enough switching speeds to rectify a THz signal. Moreover, the absence of mature theory and design equations for nano antennas makes these designs even more challenging.

This wok focuses on three major problems faced by THz rectenna devices at present. The first problem is to design and fabrication a nano antenna that can produce maximum near field intensity enhancement. The second problem is to design and fabricate a THz rectifier which has a high AC to DC conversion efficiency and is placed exactly at the hot spot to benefit from the field enhancement. Finally, the third problem is to achieve a good impedance match between the antenna and the rectifier by lowering the typically very high resistance of the rectifier.

To address the first problem, we present three solutions. Firstly, we optimize the geometric features of a 10.6 μm antenna with a gap of 50 nm to produce the maximum reported intensity enhancement. Secondly, we apply a theoretical model for travelling surface waves in gold nano antennas, in which the dispersion relation is calculated and momentum matching is ensured at the operating frequency. Consequently, free propagating electromagnetic waves can naturally couple to surface plasmon oscillations and induce traveling surface waves, thus causing a field intensity enhancement around the nano antenna. Finally, we design a matching section and incorporate a back reflector to improve the coupling to the antenna through the substrate.

Reported THz rectifiers have a major drawback: those that show good rectification ability have a very high resistance (mega ohms) thus impedance matching between the antenna (typical resistance around 100 ohms) and the diode is very difficult. Similarly the rectifiers, that have low resistance, exhibit very low rectification capability. We tackle this trade-off by fabricating a metal-insulator-metal (MIM) diode with a very small contact area (67 nm × 67 nm) to reduce its capacitance and with a very thin oxide layer (0.7 nm) to reduce its resistance. The MIM diode has been fabricated through electron beam lithography (EBL) and employs copper oxide (CuO with relative permittivity of 2.4 at 28.3 THz^2^) which has been realized through state-of-the-art atomic layer deposition technique. The very small oxide thickness results in a relatively low zero bias resistance of 500 Ω. The diode not only overcomes the tradeoff of high responsivity (measure of its rectification ability) and low resistance, but it is also exhibits the ability to rectify a THz signal without the application of any electrical source. In other words, the turn-on voltage of the diode is zero. The zero bias performance of the diode suggests that the design is highly suitable for energy harvesting applications.

With support from the US Air Force in 1964, William Brown demonstrated that wireless power transmission at microwave frequencies could power a small helicopter, thereby developing the first rectenna^3^. After Brown's invention a considerable amount of research was conducted to investigate microwave rectenna devices^4^,^5^. Infrared or optical antennas, on the other hand, have not been extensively investigated before, since they require complicated fabrication facilities like EBL, in addition to huge computational resources required to create the nano mesh. Nano antennas recently became popular due to their ability to localize incident electromagnetic fields in sub-wavelength volumes^6^,^7^. Over the past 15 years, researchers have focused on investigating the optical properties of metallic nano particles^8^,^9^,^10^,^11^,^12^, and it was observed that when a visible or infrared light impinges on an antenna's surface, it excites surface plasmon oscillations and drives the current towards the feed point of the antenna, creating a hot spot where the field intensity is enhanced. This phenomenon can be used to design antennas for visible light^12^,^13^,^14^ that have many applications, such as nano-scale imaging and spectroscopy^15^,^16^, improving solar cell efficiency^17^,^18^,^19^, and coherent control^20^,^21^. In addition, the concept is also useful for electrically tunable infrared plasmonic structures^22^ and frequency tunable THz antennas^23^.

For rectification purposes, available diodes (semiconductor based) are not fast enough to rectify THz signals. The only way to achieve THz rectification is through the tunnelling effect where electrons tunnel through a thin layer of oxide in approximately 10^−15^ sec. The rapid tunneling phenomenon makes tunnelling diodes the only suitable devices for THz rectification. This quantum mechanical effect cannot happen through insulators that are thicker than a few tens of angstroms since tunnelling probability falls off exponentially with the barrier thickness^24^,^25^. Tunneling diodes consist of a thin oxide layer sandwiched between two metal strips and are known as Metal-Insulator-Metal diodes, Metal-Barrier-Metal (MBM) diodes, or Metal-Oxide-Metal (MOM) diodes. The application of tunneling concept started in 1960s when point contact MIM diode was used for microwave detection and millimetre wave frequency mixing^25^. Point contact MIM consists of a sharp metallic tip in contact with a metal plane with insulator or air gap in between. The point contact metallic wire was usually made of tungsten and acts as an antenna to couple the electromagnetic signal, via its very sharp tip, to the oxide layer for rectification^26^. In order to avoid the problems with tungsten wire, such as the reproducibility of MIM diode, in addition to mechanical fragility of the fabricated diodes, planar MIM diode are typically used for rectification purposes at very high frequencies. Planar MIM diodes are formed by two crossed thin film metallic strips with a very thin oxide in between them. The planar MIM diode provides mechanical strength, process reproducibility and ease of integration with other devices. In this paper, we present a nano MIM diode integrated with a nano antenna for THz harvesting and rectification. The proposed device has the ability to transform a THz signal into a DC electric current without the application of any external electric source.

## Results

### Nano antenna optimization and fabrication

**Figure 1:**
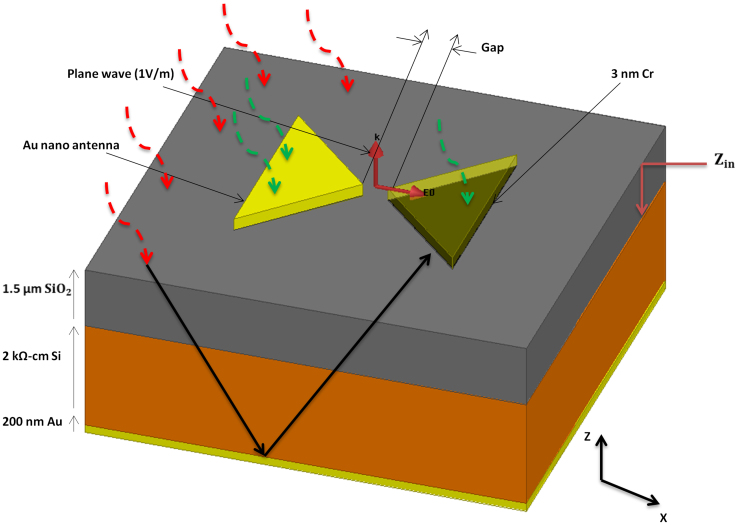
Device structure. Gold nano antenna of 100 nm in thickness is mounted on 3 nm of chromium adhesion layer. The gap size ranges from 100 nm to 0.5 nm. The substrate is made of 1.5 μm of silicon dioxide that acts as a matching section, 375 μm high resistivity silicon, and finally 200 nm of gold to function as a back reflector. Excitation is through an incident plane wave with electric field intensity of 1 V/m. *Z_in_* is the input impedance to the waves impinging on the silicon dioxide matching section (red arrows). The black arrow represents substrate coupling by the reflected waves. The green arrow represents the waves coupled to the antenna.

**Figure 2:**
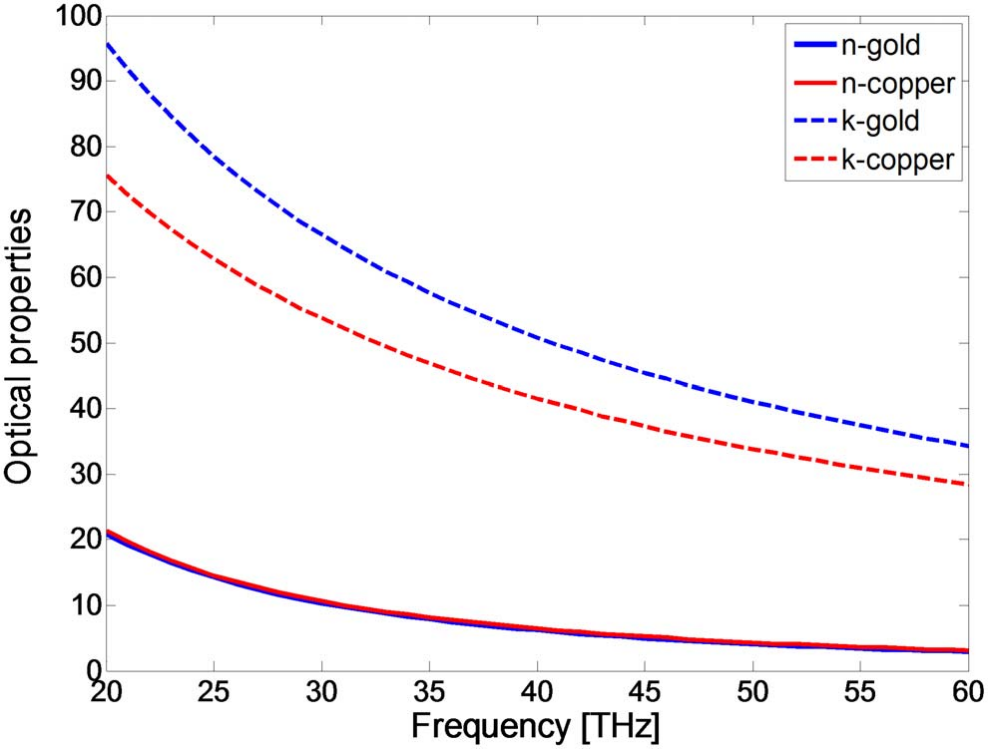
Frequency dependent optical properties of thin film gold and copper. Experimentally measured complex refractive index for thin film (75 nm), gold (blue line), and copper (red line). Gold and copper have the same frequency dependent refractive index, but the higher value of gold's extinction coefficient indicates higher absorption in THz frequencies.

**Figure 3:**
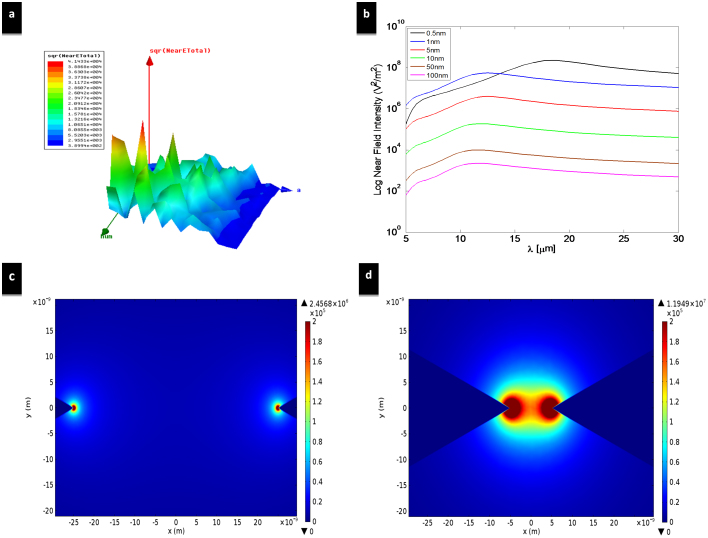
Nano antenna optimization and near-field intensity variation with gap size. (a) The near field intensity enhancement for several bowtie antennas with a gap of 50 nm is plotted against antenna length (green axis) and half bow angle (blue axis). For a gap of 50 nm, an enhancement factor of 4 × 10^4^ was achieved for 2.7 μm length and 50° bow angle. (b) Numerical simulation of the optimized bowtie dipole versus different gap sizes. The plotted relative intensity enhancement is defined as the ratio between the electric field intensity at the middle of the gap to the electric field intensity of the incident plane wave. (c) Simulated relative near field intensity enhancement for the optimized bowtie of a 50 nm gap. (d) Simulated relative near field intensity enhancement for the optimized bowtie of a 10 nm gap.

The structure studied in this work is a bowtie dipole employing gold metallization with a gap of 50 nm and it has been realized on a four layer stack up as shown in Figure 1. The first layer is a 3 nm chromium thin film used as an adhesion layer. The second is a 1.5 μm silicon dioxide matching section, employed to increase the transmission of the incoming THz signal to the silicon substrate. The third layer is a 375 μm high resistivity silicon (2 KΩ-cm) where the high resistivity reduces the substrate losses. Finally, the fourth layer is a 200 nm gold back reflector to enhance coupling to the antenna from the substrate. Detailed calculations for the stack up lengths are presented in the methods section. To model gold in the THz simulations, the experimentally measured frequency-dependent dielectric constant of gold thin film^27^ (Figure 2) has been imported to the finite element method numerical simulator (Ansys HFSS). Drude model has been intentionally avoided because it overlooks inter-band transition or absorption, which is very significant in metals at THz frequencies and can be seen in the high values of the extinction coefficient ‘*k*’ in Figure 2. The simulated structure, shown in Figure 1, is excited through a normally incident plane wave (z-axis) with an electric field intensity of 1 V/m and a linear polarization parallel to the antenna axis (x-axis). In order to overcome the huge computational resources required to simulate the nano dimensions, an equivalent problem is simulated using electric and magnetic symmetry. The electric field intensity is computed in the middle of the nano gap where simultaneous optimization of both the length and the bow's angle of a 100 nm thick nano antenna results in a relative intensity enhancement of four orders of magnitude for a 50 nm gap, as shown in Fig. 3(a). The figure demonstrates the relative near-field intensity enhancement produced by different geometries of bowtie antenna at 28.3 THz, and as shown in the figure, the maximum enhancement is produced by the bowtie that is 2.7 μm in length and with a 50° bow angle. The simulation results indicate that the antenna can achieve a relative intensity enhancement of eight orders of magnitude if a gap of 0.5 nm can be achieved, as shown in Fig. 3(b). These results are two orders of magnitude higher than the previous reports in the literature. Table 1 compares the performances of state of the art nano antennas presented in the literature in terms of field enhancement.

**Figure 4:**
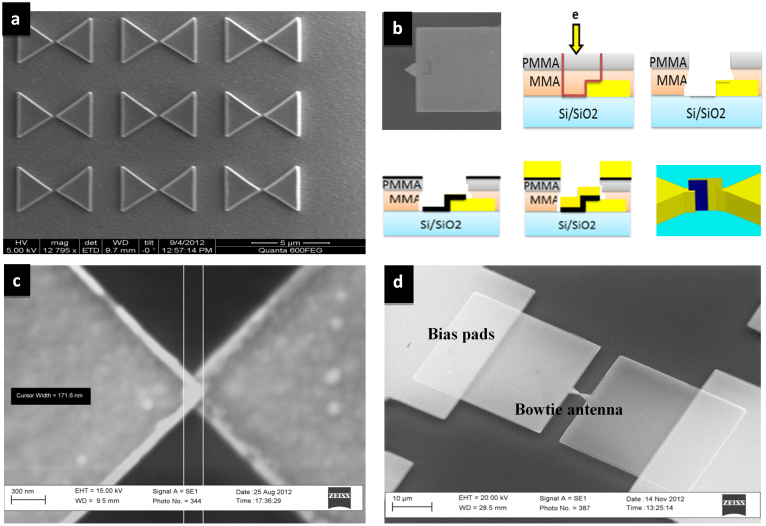
Fabrication of nano antennas and the rectenna device. (a) SEM image of the nano antenna array fabricated using EBL. (b) Overlap fabrication process: (i) First antenna arm, (ii) Second arm exposure using EBL, (iii) Removing the exposed resist using a mixture of MIBK and IPA developer with ratio of 1:3, (iv) Deposition of 0.7 nm of oxide using atomic layer deposition (ALD), (v) Second arm sputtering, (vi) Complete device after the liftoff process using acetone. (c) SEM image of the fabricated overlap. (d) SEM image of the antenna-integrated diode. The lower arm is made of 95 nm of gold over a 5 nm chromium layer, while the upper arm is made of 100 nm of copper. The tips of both arms sandwich 0.7 nm of copper oxide. Bias pads (20 μm × 32 μm) are connected to the antenna for diode bias and signal readout purposes.

To study the effect of the gap size on the value of the relative intensity enhancement, several gaps of 0.5 nm, 1 nm, 5 nm, 10 nm, 50 nm and 100 nm have been investigated with a frequency sweep of 20 to 60 THz, as shown in Fig. 3(b). Simulations results show that the increase in the gap size results in a significant decrease in the field intensity, but a very slight variation in the resonance frequency. Field enhancement increases with the decreasing gap size due to the increase of the charge concentration near the gap. Simulations have also been performed in the multi-physics simulator COMSOL to verify HFSS simulations and very similar filed enhancements have been observed in COMSOL. Fig. 3(c) and Fig. 3(d) demonstrate the simulation results from COMSOL, where the field concentrations in a gap of 50 nm and 10 nm are shown respectively. As can be seen, the value of the field enhancement decreases as the measurement point goes away from the bowtie tip. The maximum value of the enhanced field is localized exactly at the bowtie tip. Based on this observation, an overlapped rectenna device has been proposed in this work, where the bowtie arms overlap and the THz rectifier is realized in between this overlap. Even though the proposed design adds fabrication challenges, it offers two main advantages as compared to popular rectenna designs^28^. First, the overlap design takes advantage of the sharp bowtie tip which is the main reason for the field enhancement. Second, the only available path for the highly localized electromagnetic fields at the tip is through the rectifier. Fig. 4(a) shows an SEM image for an array of optimized nano antennas that is fabricated using EBL and lift-off process. The 1.5 μm silicon dioxide matching section is deposited by plasma enhanced chemical vapour deposition (PECVD). The overlap fabrication steps in addition to the fabricated rectenna device are shown in Fig. 4(b), Fig. 4(c), and Fig. 4(d). The characterization and fabrication process are described in the methods section.

### THz diode design

**Figure 5:**
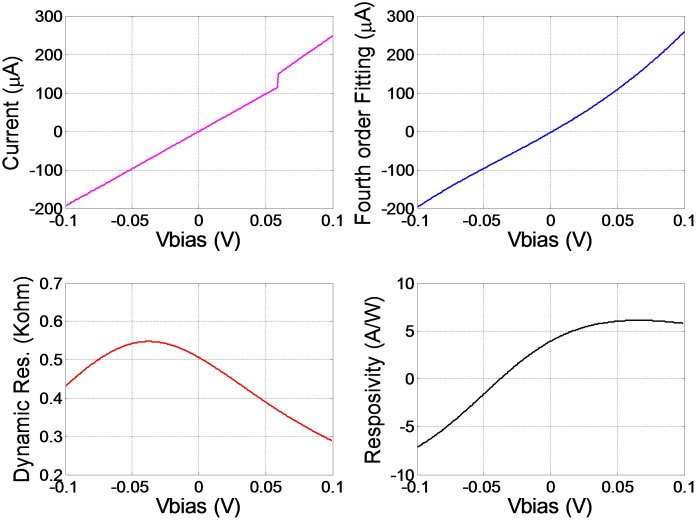
Rectenna device performance. (a) The *I-V* characteristics of the MIM diode. (b) Fourth-order fitting of the I-V curve. (c) The diode's dynamic resistance with a value of 500 Ω at zero bias. (d) Responsivity with a value of 4 A/W at zero bias and maximum value of 6 A/W at 0.1 V.

The voltage across the diode due to the flow of AC current with angular frequency *ω* takes the following form: 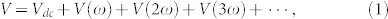
where the rectified DC voltage depends on the variation in the current across the diode with respect to the bias voltage (V_bias_)^29^: 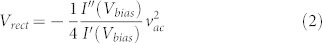
where *V_ac_* and *V_rect_* are the amplitude of the AC input signal and DC output voltage respectively. *I*′(*V_bias_*) and *I*″(*V_bias_*) are the first and the second derivative of the electric current passing through the diode at a certain bias voltage. According to equation (1), the higher the non-linearity in the diode's *I-V* characteristics, the higher the rectified voltage, thus the rectenna's responsivity ‘*S*’ is defined as: 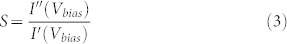
The performance of any MIM diode is determined based on three main characteristics. The first and the most important characteristic is the diode's responsivity (Eq. (3)) which is a measure of diode's rectification ability. Diode's resistance is the second important characteristic as it affects the matching between the antenna and the rectifier. The third characteristic is the diode's cut-off frequency which, in our case, is required to be in THz. 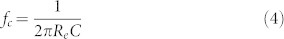


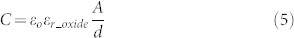
where *R_e_* is the equivalent resistance of the diode and the antenna, *C* is the MIM's capacitance, *ε_r_oxide_* and *d* are the relative dielectric constant and the thickness of the oxide respectively. *A* is the overlap or contact area. According to Eq. (4) and (5), increasing the oxide's thickness decreases the capacitance which in turn increases the cut-off frequency as well as the nonlinearity of the *I-V* curve. However, this will increase the diode's resistance, since the number of tunneling electrons decreases exponentially as the diode's thickness increases. For energy harvesting applications, the diode should be sensitive even without the application of any external electrical source. To simultaneously achieve high zero bias sensitivity and low zero bias resistance, copper oxide is proposed as the insulator for the MIM diode with a thickness of 0.7 nm and the contact area of the MIM diode has been chosen to be 67 nm × 67 nm. The diode is sandwiched between two dissimilar metals (Gold and Copper) with different work functions to allow Fermi-level gradient on both sides of the potential barrier and thus electron tunneling can occur. Gold (work function = 5.1 eV) is used for the lower antenna arm, and then 0.7 nm of copper oxide is deposited before overlapping the second antenna arm, which is made of copper (work function = 4.7 eV). Electrical testing results of the rectenna device demonstrating the THz rectifier performance are plotted in Figure 5.

After the *I-V* characteristics were measured, polynomial fitting of the fourth order was implemented to compute the rectifier's responsivity and impedance. The Au/CuO/Cu rectenna exhibits a high zero bias responsivity of 5 A/W due to the use of dissimilar metals, and a low zero bias resistance of 500 Ω and thus can be matched with the antenna.

Table 2 compares the performance of the state-of-the-art MIM diodes with the proposed design. It can be seen that previously reported MIM diodes that have high zero bias responsivities suffer from large resistances (mega ohms)^30^,^31^,^32^. Matching with the antenna in this case is extremely difficult. On the other hand, those with low resistances^33^,^34^ have very low zero bias responsivities (10^−3^ A/W) and thus demonstrating poor rectification capabilities. The proposed design, however, offers an optimum performance for both aspects, that is high zero bias responsivity (4 A/W) and a reasonably low zero bias resistance (500 Ω). In addition to diode's ability to work at zero bias, it demonstrates good rectification capability and offers a lower impedance mismatch with the designed receiving antenna (impedance ~ 100 Ω).

## Discussion

Our results show that a carefully optimized nano antenna can produce up to 8 orders of relative intensity enhancement. Morever, a CuO MIM diode is very suitable for THz rectification detection when it comes to energy harvesting applications because it is able to produce high zero bias responsivity in addition to low zero bias resistance, which allows for both THz rectification and matching between the antenna and the diode. In this section, we discuss the physical as well as mathematical reasons behind the near field enhancement in nano antennas, in addition to how infrared reception can be improved via stack up manipulation.

**Figure 6:**
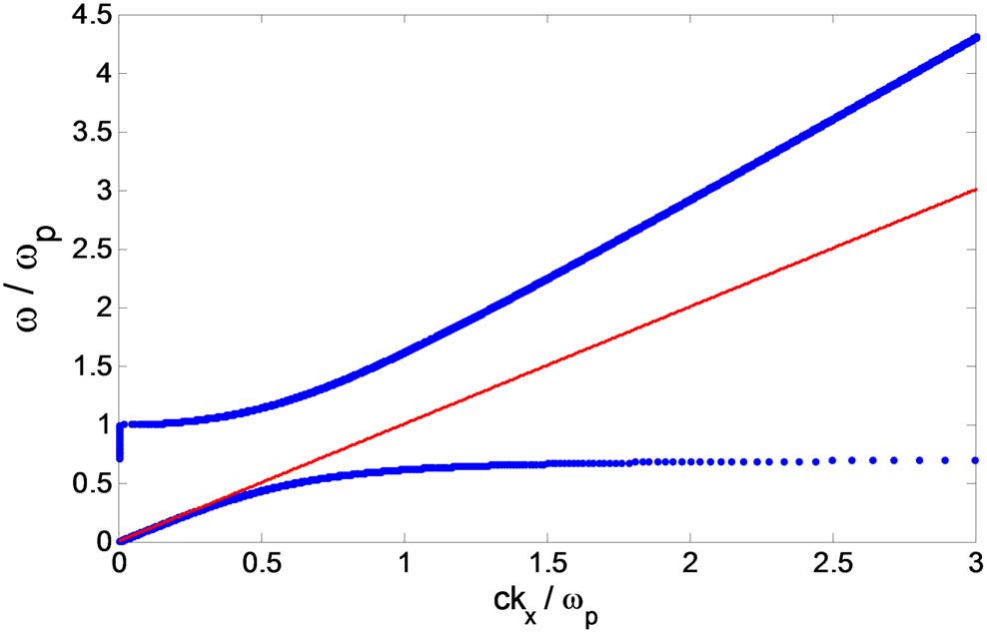
Gold Surface plasmon's dispersion relation. The blue curve shows the dispersion relation of the surface plasmons. A band gap exists from 0.7 ω_p_ to ω_p_ and there is zero group velocity at an angular frequency of 0.7 ω_p_. The dotted red line shows the free space dispersion relation. Momentum matching is achieved up to 0.3 ω_p_.

To begin with, the continuous motion of the negative free charge carriers around the positive fixed ions in plasma creates positions with high concentration of negative charge carriers and positions with low concentration of negative charge carriers. The gradient (fluctuation) of the negative charges on the surface creates electric field lines starting from points with low concentrations and ending at points with high concentrations. The induced electromagnetic fields are highly localized along the interface and exponentially decay in both the dielectric and the metal. The excitation of surface plasmon is one (but not the only) of the main reasons behind the field enhancement and localization in the nano gap. Surface plasmon propagate along the interface between the metal and the dielectric, and thus the transverse magnetic (TM) time harmonic electromagnetic waves take the following form:

- Fields in the dielectric: 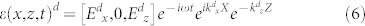

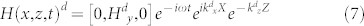
- Fields in the metal: 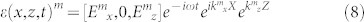

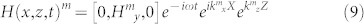
where *ω* is the angular frequency of the alternating field, *k^d^_x_* and *k^m^_x_* are the wave vectors in the *x*-direction in air and the antenna, respectively, and *k^d^_z_* and *k^m^_z_* are the decaying constants in the positive z-direction in air and in the negative z-direction in the antenna, respectively. These equations satisfy the mathematical solution of Maxwell's differential equations and also model the traveling surface waves' physical behavior, since they describe evanescent evolution with positive and negative z-directions and propagation along the interface in the *x*-direction. To ensure the continuity of the tangential electric field components and the conservation of the electromagnetic waves' momentum along the interface, the following conditions must be satisfied: 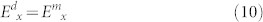

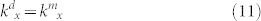
To satisfy the continuity of the normal components of the magnetic field vector and the displacement vector, the following conditions must be satisfied: 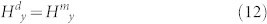

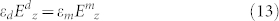
After straight forward algebra by satisfying Maxwell's second equation, 
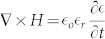
 and the Helmholtz wave equation, 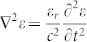, the dispersion relation can be expressed as^35^: 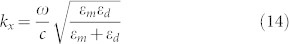
where *ε_m_* is the dielectric function of the metal, *ε_m_* = 1 − *ω_p_*^2^/*ω*^2^ and *ω_p_* is the metal's plasma frequency and is given by *ω_p_*^2^ = *Ne*^2^/*ε*_o_ *m*. Figure 6 shows the dispersion relation of a gold antenna. The red line represents the free space dispersion relation, where the angular frequency of the electromagnetic wave is proportional to its spatial frequency with a constant rate of change, which is equal to the phase or group velocity of the electromagnetic waves. The surface waves' dispersion relation, however, is not that simple. The first observation is that there is no solution for frequencies between 0.7 *ω_p_* and *ω_p_*. In other words, electromagnetic waves with angular frequencies that satisfy 0.7 *ω_p_* < ω < *ω_p_* cannot propagate as surface waves simply because there is no value for their wave vectors. The second observation is that the value of *k_x_* is constant at *ω* = 0.7 *ω_p_*. Thus, at very high values of the wave vector along the x-direction, the group velocity (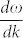) is equal to zero. For the proposed design, the dielectric is free space and the metal is gold (*ω_p_* = 1369.7 * 10^12^*r*/*s*). In this case, the interaction between light and surface plasmon is very strong. In order to couple incident electromagnetic power to travelling surface plasmon's modes, their wave vectors must be the same to conserve momentum and satisfy boundary conditions. The dispersion relation (Figure 6) shows that the momentum is conserved for side coupling of free propagating modes into surface waves that are localized at the interface in the region from 0 to 0.3 *f_p_*. Moreover, according to Stockman^36^, the group and phase velocities of electromagnetic waves propagating on the surface of a tapered cone decrease with propagation until they vanish at the pointed end. This creates a shock wave and electromagnetic energy accumulates at the end of the tapered cone or the bowtie antenna. The plasma frequencies of gold and copper can be calculated from the relation given before to be 2180 and 2610 THz, respectively. According to Figure 6, momentum matching can be achieved till 654 THz for gold and 783 THz for copper. For energy harvesting applications from waste heat, the targeted frequency range is from 27 to 150 THz, thus coupling between incident infrared and surface waves is guaranteed. For frequencies higher than 0.3 *f_p_* at which there is momentum mismatch, electromagnetic energy can still be coupled to the antenna by the use of gratings or prisms.

Finally, due to symmetry, a planar antenna in air radiates equally on both sides. This symmetry can be broken by the presence of a substrate on one side, which in turn changes the phase velocity and current distribution on the antenna's surface. In this case, the antenna radiates most of its power to the substrate side^37^. Due to reciprocity, an antenna on a substrate couples more energy from the substrate side where the ratio between them is given by: 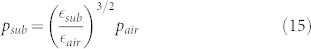
where 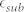 is the substrate's relative permittivity. At 28.3 THz, the effective permittivity of a silicon substrate is 11.6^28^. The electromagnetic power coupled to the antenna from the substrate side is thus 40 times that of what is coupled from the air side. To exploit this property, a matching section is utilized to improve the transmission from air to silicon. In addition, a back reflector is employed to reflect the signal coupled in silicon substrate to the antenna. A silicon dioxide layer of 1.5 μm is added between the air and the silicon substrate to act as a matching section at 10.6 μm. The matching section improves the transmission to the substrate to 75% from 45%, as described below. Intrinsic impedance of silicon has a value of 120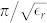 and thus transmission from air to silicon is given by: 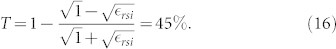
Equation 16 shows that when an electromagnetic wave is incident on a silicon surface, 55% is reflected and 45% is transmitted, in other words around half of the power is lost. If a 1.5 μm silicon dioxide *(SiO*_2_) layer is added as a matching section between the air and Si, transmission to the Si substrate can be improved.

The input impedance for electromagnetic waves incident from air to *SiO*_2_ over the silicon substrate is given by: 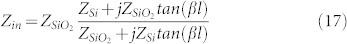
where β = 2π/λ, 
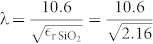
 Thus, the effective wavelength of light in *SiO*_2_ is 7.2 μm. 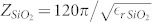, 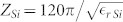. By substituting these values in Eq. (17), we get Z_in_ = 1.7*η_0_ and 
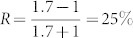
. In other words, adding 1.5 μm of *SiO*_2_ increases transmission by 30%. The silicon dioxide layer also functions as isolation between the diode and the substrate to avoid formation of a Schottky diode. As mentioned above, ideally, all the power coupled to Si substrate should be coupled to the antenna from the substrate side through the back reflector. It is worth mentioning here that Eq. (17) ignores the perturbation introduced by the antenna itself to the incident fields. Nonetheless, the measures taken here to enhance the captured fields can be considered as preliminary optimization steps. Detailed antenna design techniques can enable even better optimization.

The SiO_2_ matching section can be added to the silicon wafer using several techniques, such as sputtering, atomic layer deposition (ALD) or plasma enhanced chemical vapor deposition (PECVD). ALD produces the best uniformity of a thin film, but it is time consuming. PECVD produces good uniformity at a high deposition rate with a precise thickness, so it is chosen to deposit the matching section.

For the back reflector, the wafer is coated with a gold layer of thickness four times that of the skin depth at the operating frequency. The electron's relaxation time is given by: 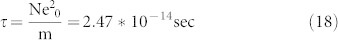
where *e* = 16*10^−19^ *c*, *m* = 6.1*10^−31^ *kg*, *N* = 5.9*10^28^ *m*^−3^, *f_p_* = 2.18*10^15^ Hz. For frequencies comparable to the damping frequency, *γ*, the skin depth is given by: 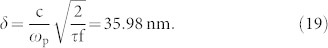
The back reflector is 200 nm, which is around five times that of the skin depth of gold at 28.3 THz, and is enough to ensure total reflection from the back of the substrate. The back reflector has been added using an electron beam evaporator.

## Methods

### Fabrication process and device testing

Electrode patterning was done by electron-beam lithography using a modified ZEISS scanning electron microscope (SEM) with a LaB6 cathode. The cathode was operated at an accelerating voltage of 30 kV. The exposure current was 11 pA. Raith Elphy Quantum software was used to manipulate the electron beam. A bilayer resist process was used. The bottom layer was a 350 nm copolymer methyl methacrylate-methacrylic acid (MMA-MAA) baked for 10 min. on a hotplate at 180°C. The top layer was 150 nm 495-K polymethyl methacrylate (PMMA) baked for 10 min on a hotplate at 180°C. The electrodes were exposed in a 100 μm × 100 μm write field at an area dose of 300 μC/cm^2^ and 1 μs of dwell time. The resist was post exposure developed for 30 sec. in a 1:3 mixture of methylisobutylketone:isopropanol (MIBK:IPA). After patterning the first MIM electrode along with one bowtie antenna arm, a 5 nm thick film of Chromium (Cr) followed by a 95 nm thick film of gold (Au) were deposited using DC sputtering at 150 W of power at a chamber base pressure of 7 × 10^−7^ Torr and Argon (Ar) pressure of 3 mTorr. Liftoff was then performed by removing all the excess metal and ending with one bowtie antenna arm and an Au electrode. Immediately after patterning the second bowtie antenna arm, 0.7 nm of CuO was deposited using atomic layer deposition at 150 W of power and Ar pressure of 3 mTorr. The second MIM diode electrode and the second bowtie antenna arm were then formed from a 100 nm thick copper (Cu) film that was deposited using DC sputtering at 150 W of power at a chamber base pressure of 7 × 10^−7^ Torr and Argon (Ar) pressure of 3 mTorr. Finally, lift off was performed. The *I-V* characteristics, of the fabricated MIM diodes, were measured at room temperature using an HP B1500 semiconductor device analyzer with a probe station setup. Fabrication steps for the overlapped second antenna arm are shown in Figure 4. A 1.5 μm silicon dioxide matching section was deposited using PECVD at pressure of 1000 mT, RF power of 20 W, table temperature of 300 C, and deposition rate of 66 nm/min.

## Author Contributions

M.N.G. designed and simulated the nano antennas and the rectenna device, wrote the manuscript, post processed and interpreted the measured data, fabricated the nano antenna array and finally did the theoretical analysis in the discussion section. M.A. co-designed and fabricated the rectenna device. A.S. supervised the project and contributed to the general concept, validation of design and results and preparation of the manuscript.

## Figures and Tables

**Table 1:** State-of-the-art near-field intensity enhancement by different nano antennas

Reference	Resonance wavelength (nm)	Gap size (nm)	Relative intensity enhancement (orders of magnitude)	Antenna structure
**Kottmann et al. 2001^38^**	329	No gap	5	Irregular triangle
**Sundaramurthy et al. 2005^39^**	856	16	4	Bowtie
**Fischer et al. 2008^40^**	820	30	2	Bowtie
	760	30	3	Dipole
**Seo et. al 2009^41^**	3*10^6^	70	3	Slit
**McMahon et al. 2009^42^**	900	0.25	5	Dimer
**McMahon et al. 2010^6^**	570	0.5	5	Bowtie
**Feichtner et al. 2012^7^**	647	10	3	Split ring + 2 wire antenna
**Chen et al. 2012^43^**	830	30	2	Bowtie
**Palma et al. 2013^44^**	75000	Cavity	4	Square patch over a dielectric layer
**This work**	10600	0.5–1	8	Bowtie
		10	6	
		50	4	

**Table 2:** State-of-the-art MIM diode responsivity

Reference	Type of MIM	Maximum responsivity (*V*^−1^)	Zero bias responsivity (*V*^−1^)	Zero bias resistance (ohm)	Oxide thickness (nm)
**Hoofring 1989^45^**	Thin film Ni-NiO-Au (0.64 μm^2^)	5.5	2.8	–	2.2
**I. Wilke, et. al 1994^33^**	Ni-NiO-Ni (0.0576 μm^2^)	1.6	--	100	4
**M. Abdel-Rahman et al. 2004^46^**	Thin film Ni-NiO-Ni (0.075 μm^2^ and 0.0014 μm^2^)	2.75 and 1.65 respectively	--	180	3.5
**Esfandiari 2005^47^**	Thin film Ni-NiO-Pt (0.0025 μm^2^)	−13	−3	--	2
**S.Krishnan et al. 2008^30^**	Thin film Ni-NiO-cr/Au (1 μm^2^)	5	1	500 K	3
**Choi et. al 2010^48^**	polysilicon-SiO2-polysilicon(60 nm^2^)	−31	12	--	1.38
**Dagenais et al. 2010^32^**	Thin film polysilicon-SiO2-Au (0.35 μm^2^)	−14.5	2.5	120 M	60
**Bean et al. 2011^49^**	Thin Al-AlOx-Pt (0.5625 μm^2^)	−2.3	0.5	220 K	0.6
**Zhang et al. 2013^31^**	Ni-NiO-Cu (0.008 μm^2^)	7.3	--	1.2 M	2 – 12
**Kinzel et al. 2013^34^**	Al-Al2O3-Pt (0.008 μm^2^)	0.03	1.24*10^−3^	124.6	2
**Zhu et al. 2013^50^**	Graphene-Air-Graphene	0.24	0.12	--	--
**This work**	Cu-CuO-Au (0.0045 μm^2^)	6	4	505	0.7
